# Development of Chemical Tools to Monitor Human Kallikrein 13 (KLK13) Activity

**DOI:** 10.3390/ijms20071557

**Published:** 2019-03-28

**Authors:** Natalia Gruba, Ewa Bielecka, Magdalena Wysocka, Anna Wojtysiak, Magdalena Brzezińska-Bodal, Kamila Sychowska, Magdalena Kalińska, Małgorzata Magoch, Aleksandra Pęcak, Katherine Falkowski, Magdalena Wiśniewska, Laura Sąsiadek, Karolina Płaza, Eileen Kroll, Anastasija Pejkovska, Maren Rehders, Klaudia Brix, Grzegorz Dubin, Tomasz Kantyka, Jan Potempa, Adam Lesner

**Affiliations:** 1 Faculty of Chemistry, University of Gdansk, 80-308 Gdansk, Poland; natalia.gruba@ug.edu.pl (N.G.); magdalena.wysocka@ug.edu.pl (M.W.); anna.wojtysiak@phdstud.ug.edu.pl (A.W.); magdalena.brzezinska@phdstud.ug.edu.pl (M.B.-B.); kamila.sychowska@gmail.com (K.S.); 2 Malopolska Centre of Biotechnology, Jagiellonian University, 30-387 Krakow, Poland; ewa.bielecka@uj.edu.pl (E.B.); m.magoch@gmail.com (M.M.); aleksandra.pecak@gmail.com (A.P.); katherine.falkowski@doctoral.uj.edu.pl (K.F.); wisienka.magdalena@gmail.com (M.W.); lmsasiadek@gmail.com (L.S.); grzegorz.dubin@uj.edu.pl (G.D.); tomasz.kantyka@uj.edu.pl (T.K.); 3 Faculty of Biochemistry, Biophysics and Biotechnology, Jagiellonian University, 30-387 Krakow, Poland; magda.kalinska@uj.edu.pl (M.K.); plaza.karolina@gmail.com (K.P.); 4 Department of Life Sciences and Chemistry, Jacobs University Bremen, 28759 Bremen, Germany; ek.eileenkroll@gmail.com (E.K.); anja.pejkovska@gmail.com (A.P.); m.rehders@jacobs-university.de (M.R.); k.brix@jacobs-university.de (K.B.); 5 Broegelmann Research Laboratory, Department of Clinical Science, University of Bergen, 5020 Bergen, Norway; 6 School of Dentistry, University of Louisville, Louisville, KY 40202, USA

**Keywords:** KLK13, substrate specificity, combinatorial chemistry, fluorogenic substrate, activity-based probe

## Abstract

Kallikrein 13 (KLK13) was first identified as an enzyme that is downregulated in a subset of breast tumors. This serine protease has since been implicated in a number of pathological processes including ovarian, lung and gastric cancers. Here we report the design, synthesis and deconvolution of libraries of internally quenched fluorogenic peptide substrates to determine the specificity of substrate binding subsites of KLK13 in prime and non-prime regions (according to the Schechter and Berger convention). The substrate with the consensus sequential motive ABZ-Val-Arg-Phe-Arg-ANB-NH_2_ demonstrated selectivity towards KLK13 and was successfully converted into an activity-based probe by the incorporation of a chloromethylketone warhead and biotin bait. The compounds described may serve as suitable tools to detect KLK13 activity in diverse biological samples, as exemplified by overexpression experiments and targeted labeling of KLK13 in cell lysates and saliva. In addition, we describe the development of selective activity-based probes targeting KLK13, to our knowledge the first tool to analyze the presence of the active enzyme in biological samples.

## 1. Introduction

Human kallikreins (KLKs) include 15 serine proteases with trypsin- or chymotrypsin-like specificities [1]. KLKs are characterized by diverse tissue distributions and developmental stage expression and, as such, have diverse biological functions [2]. Deregulated expression of KLKs is characteristic for pathological conditions and is implicated in hypertension, inflammation, neurodegenerative disorders and cancer [3,4,5,6]. Accumulating evidence associates KLKs with different malignancies which points to their potential as biomarkers. KLK3, better known as prostate-specific antigen (PSA), is widely used in detecting and monitoring prostate cancer progression [7,8], but other kallikreins will likely prove useful to monitor a variety of cancers.

In homeostasis, human kallikrein 13 (KLK13) is primarily expressed in the testis, prostate, breast, salivary glands, esophagus and the cervix [9,10,11,12] and is associated with certain skin pathologies, including psoriasis vulgaris and atopic dermatitis [13]. Moreover, KLK13 is expressed at high levels in some types of salivary gland tumors [14]. Furthermore, it has been reported as an independent, favorable prognostic marker in breast [15] and ovarian cancers [16] and has potential clinical utility as a prediction marker of gastric cancer cells’ response to chemotherapy [17]. As such, deciphering the biological pathways involving KLK13 is of particular interest, and suitable tools to detect, label, monitor and quantify its proteolytic activity are in great demand.

Proteins can be detected with high sensitivity even in very complex physiological samples using immunolabeling. However, such methods are only rarely able to detect the level of activity, which may differ significantly from the amount of an enzyme itself. Monitoring the activity of individual proteases in complex samples is difficult, though possible, with the application of specific probes labeling an enzyme of interest, based on its substrate preference. KLK13 substrate specificity was investigated by Borgoño and colleagues [18]. Using 7-amino-4-carbamoylcoumarin (AMC)-based positional scanning combinatorial libraries of peptide substrates they profiled the P_4_-P_1_ (nomenclature according to Schechter and Berger [19]) subsite specificity of KLK13. They observed that KLK13 prefers arginine at the P_1_ and P_3_ positions, while aliphatic and aromatic sidechains are preferred at the P_4_ and P_2_ positions (Table 1). Additional insight into the primed subsite specificity of KLK13 was provided by Andrade and colleagues [20] who used a fluorescence-quenched peptide substrate corresponding to the specificities of KLK1 and KLK6 (ABZ-KLRSSKQ-EDDnp) [21] to design P_3_-P_2_′ spanning sub-libraries which they used to profile the specificity of KLK13. These experiments confirmed a high preference of KLK13 for basic residues (Arg preferred over Lys) at P_1_, but no clear specificity for P_3_, P_2_, P_1_′ subsites was established (Table 1) [20]. These latter data, however, are disputable due to the design of the substrate (see Section 3). Together, prior studies provide a partial, although incomplete, overview of KLK13 substrate preference, especially concerning profiling of the primed sites. More importantly, however, they have not translated into specific tools to monitor protease activity, particularly in complex tissue samples.

In the present work, we re-evaluate the prior specificity profiling results of KLK13 at non-primed positions using substrates of a different design. We extend the profiling at primed sites with extensive positional scanning and provide a sensitive and selective internally quenched substrate with the sequential motif encompassing the most efficiently recognized residues at the P_4_-P_3_′ subsites. The substrate is converted into an inhibitor and further into an activity-based probe, which allows detection of KLK13 in complex biological samples.

## 2. Results

**Figure 1:**
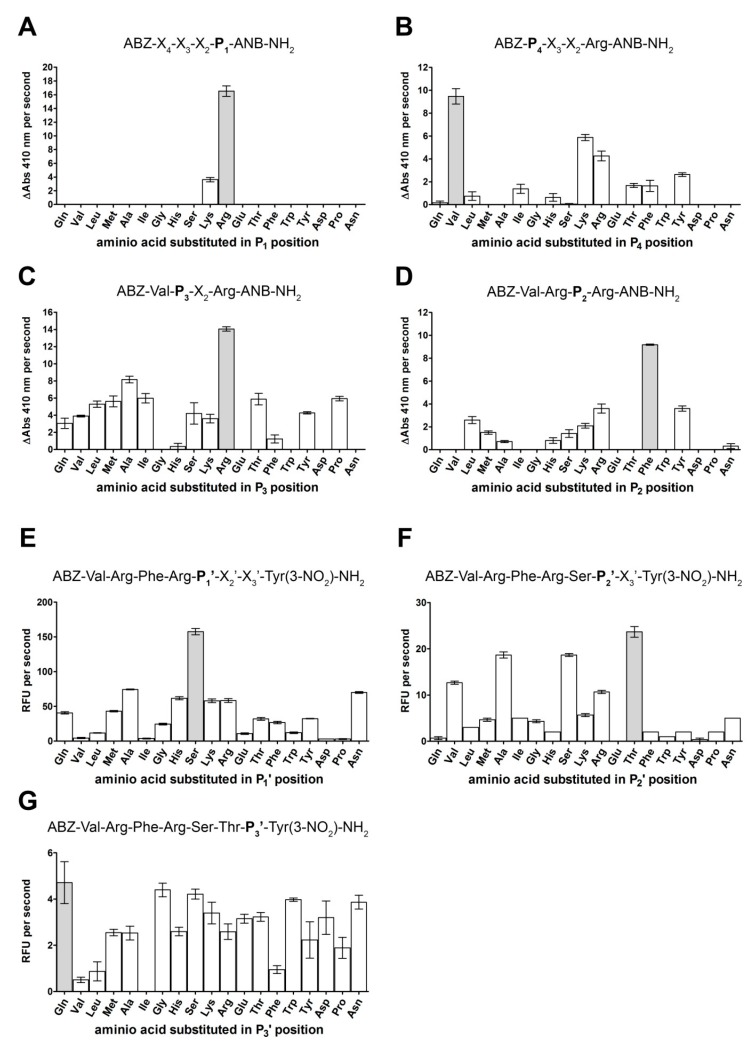
Substrate specificity of KLK13. Non-primed specificity was determined by deconvolution of ABZ-X_4_-X_3_-X_2_-Arg-ANB-NH_2_ libraries. Each sub-library (*X*-axis) was treated with KLK13 in 50 mM Tris-Cl (pH 7.5) containing 1 mM EDTA and 0.5 μM heparin at 37 °C. Absorbance of released 5-amino-2-nitrobenzoic acid (ANB) was determined at 410 nm. Panel order reflects the deconvolution of the library, with P_1_ resolved first (**A**), followed by P_4_-P_2_ analysis (**B**–**D**). Primed subsites’ specificity was determined by deconvolution of the ABZ-Val-Arg-Phe-Arg-X_1_′-X_2_′-X_3_′-Tyr(3-NO_2_)-NH_2_ libraries (**E**–**G**). Each sub-library was treated with the enzyme in 50 mM Tris-Cl (pH 7.5) buffer containing 1 mM EDTA and 0.5 μM heparin at 37 °C. The fluorescence of released ABZ-peptide (excited at 320 nm) was monitored at 450 nm.

To devise optimal substrates and activity-based probes suitable for monitoring the activity of KLK13 we thoroughly profiled the substrate specificity of the enzyme. First, we assessed the specificity at the P_4_-P_1_ subsites using a methodology resembling that of Borgoño and colleagues [18], however, using different reporter groups and a different deconvolution scheme. Such an approach and comparative analysis allowed us to minimize the effect of substrate design on the profiling outcome. We used a fluorescence-quenched library of the general structure of ABZ-X_4_-X_3_-X_2_-P_1_-ANB-NH_2_ to profile the P_1_ residue specificity. In this library, each of the 19 sub-libraries contained a defined proteinogenic amino acid residue at P_1_ while X_4_-X_2_ positions contained equimolar mixtures of those residues. The library was deconvoluted using KLK13 by monitoring the absorbance (410 nm) of released ANB-NH_2_ to ensure specific hydrolysis at the P_1_-ANB ester bond (unlike fluorescence which is released no matter which bond is hydrolyzed). Using such a library, arginine was selected as the preferred residue at the P_1_ site for KLK13, followed by lysine, characterized by less than 30% activity of the arginine containing sub-library. The other sub-libraries, containing different residues at P_1_, were not hydrolyzed (Figure 1A).

Subsites other than P_1_ were profiled, starting from P_4_, to better explore the likely weaker interactions at this position before P_3_ and P_2_ were deconvoluted. A library of a general structure ABZ-P_4_-X_3_-X_2_-Arg-ANB-NH_2_ was prepared by the mix and split method [22] and deconvoluted with KLK13 while monitoring ANB-NH_2_ release. Valine was significantly preferred at the P_4_ subsite, with lysine and arginine also accepted, but with lower preference compared to Val (Figure 1B). Bulky sidechains of tyrosine and phenylalanine were accepted, as well as threonine, isoleucine, leucine and histidine. Sub-libraries containing other residues at P_4_ were not hydrolyzed at a detectable level. Val was fixed at the P_4_ subsite and the ABZ-Val-P_3_-X_2_-Arg-ANB-NH_2_ library was synthetized and deconvoluted by monitoring ANB-NH_2_ release. Arginine was the most preferred residue at P_3_, but the protease selectivity at this subsite was relatively low since many tested residues were recognized and the relevant sub-libraries were hydrolyzed with comparable efficiencies (Figure 1C). Only glutamic acid, aspartic acid, asparagine, glycine and tryptophan were excluded at P_3_ while substrates containing histidine at this subsite were hydrolyzed only with negligible efficiency. Position P_3_ was fixed with arginine and the ABZ-Val-Arg-P_2_-Arg-ANB-NH_2_ library was deconvoluted. Phenylalanine was preferred at the P_2_ position followed by tyrosine and arginine (Figure 1D). Lysine, leucine, methionine and serine were also recognized with notable efficiency while histidine, alanine and asparagine containing substrates were hydrolyzed poorly. Other tested residues were not tolerated at the P_2_ position. Overall, the non-primed subsite specificity profiling of KLK13 allowed identification of a kinetically preferred substrate: ABZ-Val-Arg-Phe-Arg-ANB-NH_2_ (Substrate **1**).

For profiling of primed subsites of KLK13, the peptidyl part of the favorable non-primed substrate was incorporated into fluorescence-quenched substrate libraries of the following general formula: ABZ-Val-Arg-Phe-Arg-X_1_′-X_2_′-X_3_′-Tyr(3-NO_2_)-NH_2_ where Tyr(3-NO_2_)-NH_2_ quenches the fluorescence of ABZ. The P_1_′ library was evaluated first, by monitoring the fluorescence increase at 450 nm in the presence of the protease. Serine was selected at P_1_′ over all other tested residues, but the specificity of the protease at the evaluated subsite was relatively relaxed since many other residues were also accepted, albeit with lower efficiency compared to serine (Figure 1E). Serine was fixed at the P_1_′ position and the P_2_′ library was deconvoluted with KLK13. Small sidechains containing amino acids including threonine, serine, alanine and valine were preferred at the P_2_′ subsite in comparison to other residues (Figure 1F). Glutamic acid, aspartic acid and glutamine containing substrates were almost resistant to proteolysis. Bulky, aromatic sidechains were also disfavored. Threonine was fixed at the P_2_′ position and P_3_′ preference was evaluated. KLK13 exhibited almost no preference at this subsite (Figure 1G). Only isoleucine, valine, leucine and phenylalanine were disfavored. Glutamine was fixed at the P_3_′ position to yield a kinetically preferred substrate ABZ-Val-Arg-Phe-Arg-Ser-Thr-Gln-Tyr(3-NO_2_)-NH_2_ (Substrate **2**). These observations were validated, by re-synthesis of the optimized substrates, differing only at the P_3_′ position (Gln, Glu, Gly, Ser, respectively). Kinetic parameters of the hydrolysis were determined and confirmed limited selectivity of KLK13 at P_3_′ position. Although the P_3_′-Gln substrate was preferred, others were recognized comparably, with a notable difference for the charged Glu residue, which was hydrolyzed with approximately 3-fold lower efficiency (Table 2).

While deconvolution of non-primed libraries is unambiguous in terms of the cleavage site (only hydrolysis of P_1_-ANB-NH_2_ ester bond, but not other peptide bonds within the substrate results in absorbance increase), the results of primed sites’ deconvolution may be complicated by secondary cleavages (fluorescence is released no matter which peptide bond within the substrate is hydrolyzed). First, the ambiguity may result from the fact that two arginine residues are present within the substrate (note the primary specificity of KLK13 for arginine), and second, the selection of primed subsites may result in a shift of the major cleavage site. To verify if either of the above was the case, the scissile peptide bond was identified in each successive step of primed library deconvolution. To this end, the reaction products were analyzed with fluorescence detected RP-HPLC coupled to MS. In each case only a single and identical fluorescent peptide was identified, corresponding to ABZ-Val-Arg-Phe-Arg-OH (detected MW_exp_ = 695.8 Da) which signified that during deconvolution each X_n_ subsite truly corresponded to the P_n_′ subsite (Supplementary Figure S1; any shifted cleavages would result in additional peaks in the HPLC chromatogram). In particular, ABZ-Val-Arg-Phe-Arg-Ser-Thr-Gln-Tyr(3-NO_2_)-NH_2_ was exclusively hydrolyzed at the Arg-Ser peptide bond. No detectable hydrolysis at the Arg-Phe peptide bond signifies the importance of subsites other than P_1_ in the determination of KLK13 specificity.

To determine the sensitivity of identified substrates in detecting KLK13 activity, decreasing amounts of the enzyme were reacted with a constant amount of the substrate, and substrate hydrolysis was recorded. Detectable fluorescence increase was still observed with substrate **1** at 1.56 nM concentration of KLK13. Substrate **2** provided even more sensitive detection. Fluorescence increase was still detectable at 48.8 pM enzyme concentration (Supplementary Figure S2A,B). We also determined the specificity constants which were over 5 × 10^6^ M^−1^·s^−1^ for substrate **1** and nearly 10 × 10^6^ M^−1^·s^−1^ for substrate **2** (Table 2).

Having identified a sensitive substrate for KLK13, we re-evaluated the pH optimum of the protease activity. The proteolytic activity against substrate **1** was tested using a range of buffers with overlapping pH in the range of 3 to 10. pH 9 was identified as optimal for processing of substrate **2** by KLK13 (Supplementary Figure S2C). However, the activity dropped sharply above this value suggesting deprotonation of the catalytic histidine, while the low pH limit of activity was determined by pH 6, likely reflecting the protonation of the Asp residue in the catalytic triad.

**Figure 2:**
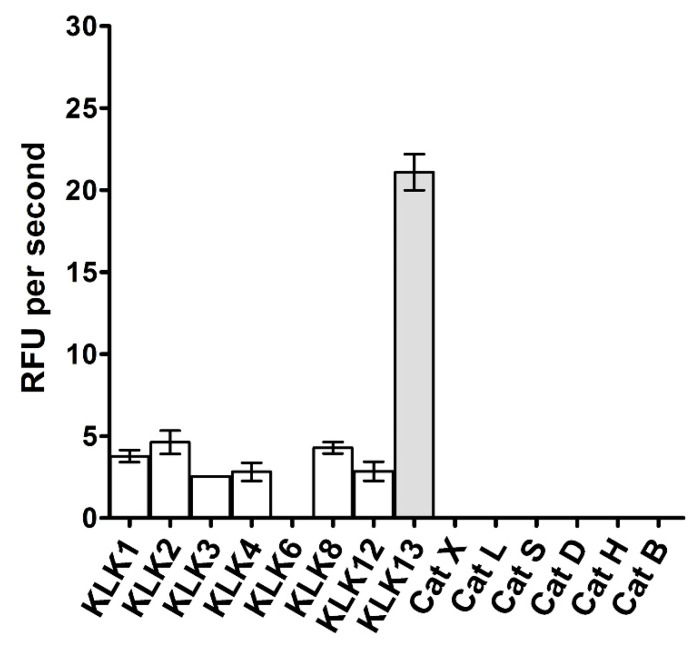
Selectivity of the optimized substrate 2 (ABZ-Val-Arg-Phe-Arg-Ser-Thr-Gln-Tyr(3-NO_2_)-NH_2_). Activity of selected kallikrein family proteases (KLK1, KLK2, KLK3, KLK4, KLK6, KLK8, KLK12, KLK13) and cathepsins (CatX, CatL, CatS, CatD, CatH, CatB) against the tested substrate was monitored at equal enzyme concentrations in the KLK13-optimized buffer. The fluorescence of the released ABZ-Val-Arg-Phe-Arg fragment was monitored at excitation and emission wavelengths 320 and 450 nm, respectively.

We next asked what the specificity of the most sensitive substrate **2** was in terms of recognition by proteases other than KLK13. To this end, we tested hydrolysis of substrate **2** by a number of kallikreins and cathepsins. KLK13 exhibited the highest activity towards the substrate; however, substrate **2** was also cleaved by a number of other kallikreins although with significantly lower efficiency, not exceeding 20% of the activity of KLK13 (Figure 2). None of tested cathepsins hydrolyzed substrate **2**.

**Figure 3:**
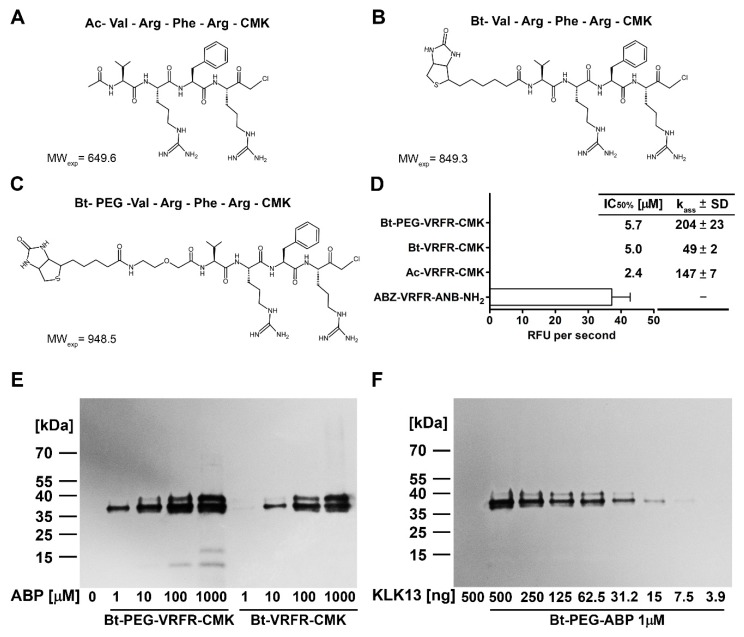
Structure and activity of the KLK13 activity-based probes. (**A**–**C**) Chemical structures of the optimized compounds developed for KLK13; (**D**) Inhibitory activity of activity-based probes against KLK13. Residual activity of KLK13 was determined in triplicates using Abz-VRFR-ANB-NH_2_ after preincubation with tested ABPs. Kinetic parameters (k_ass_ (M^−1^·s^−1^)) were determined by the progress curves method and are shown as mean ± SD from three replicates. The IC_50_ values were determined by linear regression to the initial fragment of the KLK13 activity vs concentration of the given inhibitor curve; (**E**) Biotin-conjugated ABPs specific for KLK13 were tested for protease detection in Western blot analysis. 250 ng of KLK13 was incubated with ABPs at indicated concentrations in TNET buffer for 1 h at 37 °C. ABP with a polyethylene glycol (PEG) linker showed higher sensitivity in comparison to the shorter version of the probe, allowing for KLK13 detection at 1 µM concentration and better discrimination between pro- and active KLK13 (observed as the higher molecular weight band); (**F**) Detection limit of KLK13 at an optimized probe concentration. Twofold serial dilutions of KLK13 in the range of 500 to 4 ng were incubated with 1 µM Bt-PEG-VRFR-CMK ABP in TNET buffer for 1 h at 37 °C. Labeling was detected by Western blot. The detection limit is estimated at 15 ng of purified KLK13.

Sensitive substrates allow detection of protease activity in liquid samples, but it is often advantageous to visualize the activity on solid supports (e.g., Western blot) or with high spatial resolution (e.g., microscopy). Substrate-like activity-based probes have been demonstrated to serve both these purposes. The KLK13 substrate devised in this study, (**1**), was converted to an inhibitor by inclusion of a chloromethylketone (CMK) warhead, and further into an activity-based probe (ABP) by attachment of a biotin tag linked on a varied length spacer (Figure 3A–C). As expected, all compounds inhibited KLK13 (Figure 3D), although the biotin extension slightly negatively affected the kinetics of inhibition (Figure 3D). In addition, the phosphonate warhead was evaluated, however, such inhibitors were poorly active and thus were discontinued early in the study.

To determine the detection limit and to establish the optimal concentration of the probe we analyzed the concentration-dependency of ABP labeling. Increasing concentrations of the probe were incubated with 250 ng of KLK13 and ABP binding was analyzed by Western blot. Clear labeling was detected using 1 µM concentration and increased in intensity up to 1 mM. Nonetheless, only 1 µM concentration allowed specific detection of the active protease while higher probe concentrations co-labeled the residual proKLK13 present in the sample (Figure 3E). Therefore, 1 µM ABP was used in all the following assays. To determine the detection limit in terms of protease concentration, the probe was incubated with decreasing concentrations of KLK13. Western blot analysis demonstrated that as little as 15 ng of KLK13 was detectable in the test conditions (Figure 3F).

**Figure 4:**
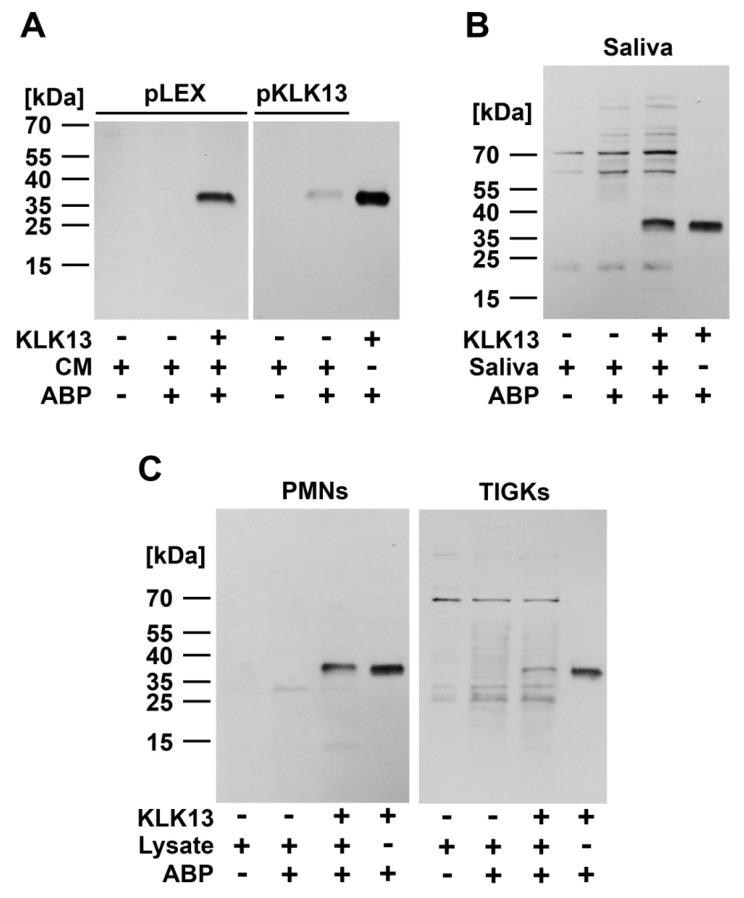
Bt-PEG-VRFR-CMK detection of KLK13 in biological samples. Effectiveness of the KLK13 probe was tested in the background of different media and cell lysates using Western blot. (**A**) Conditioned culture media (CM) of *Leishmania* Expression System (LEXSY) strains stably transfected with either empty pLEX or pLEXpKLK13 plasmids were preincubated with or *w*/*o* 1 µM KLK13 probe for 1 h at 37 °C. KLK13 was detected in the presence of ABP only in the media from cells transfected with pKLK13 and in the media of empty pLEX culture spiked with 250 ng KLK13; (**B**) Saliva was freshly collected from healthy volunteers. Diluted samples were either vehicle-treated or spiked with 250 ng of KLK13 and incubated with or without addition of 1 µM specific KLK13 probe. Signal corresponding to KLK13 was detected exclusively in the KLK13-spiked sample in the presence of ABP; (**C**) 30 µg of PMNs RIPA buffer cell lysate and 5 µg of TIGK cell lysate were either vehicle-treated or spiked with 250 ng of KLK13 and were incubated with or without the addition of 1µM specific KLK13 probe. Signal corresponding to the KLK13 molecular weight was detected in the KLK13-spiked samples in the presence of ABP. In addition, a lower-molecular weight band of unknown origin was detected in probe treated cell lysate.

The applicability of ABPs for detection of KLK13 in biological samples was initially tested using culture supernatants of *Leishmania tarentolae* expressing the recombinant protease. Conditioned media from strains transfected with the empty plasmid and the non-transfected parent strain were used as controls. A clear band corresponding to KLK13 was detected by Western blot only in the expressing strain, but not in the control supernatants, confirming the specific labeling of KLK13 (Figure 4A). A supernatant from the KLK13-non-expressing strain displayed a clear band exclusively in the KLK13-spiked sample, again demonstrating specific labeling.

To further test the ability of the ABP to detect KLK13 in even more complex biological fluids, we analyzed saliva and cell lysates of human blood neutrophils (PMN) and TIGK (gingival keratinocyte-derived, immortalized cell line). Relatively low background was present, mainly an unspecific signal found even in non-labeled samples likely signifying naturally biotinylated proteins. No KLK13 was detected in the saliva or TIGK samples, thus demonstrating that either the enzyme concentrations were below the detection limit or that KLK13 was absent in those samples. A similar pattern was true for PMN lysates, a weak intensity lower-molecular weight band was detected in labeled samples, although its origin is unknown. The KLK13 specific signal was clearly detected only in KLK13 spiked samples. Overall, this demonstrates that the probes elaborated in this study are suitable for detecting KLK13 activity even in complex biological fluids (Figure 4B,C).

## 3. Discussion

The kallikrein activome has been the subject of extensive investigation recently because monitoring KLK involvement in signaling, cancer progression and metastasis is of great importance for biomarker development and cancer pathophysiology. Therefore, development of kallikrein-directed methods and approaches is urgently needed. Here we have described a combinatorial library approach to evaluate the substrate specificity of KLK13, an enzyme involved in epithelial regulation. Unlike previous studies, our approach allowed an unbiased determination of non-primed (P_4_-P_1_) and primed (P_1_′-P_3_′) subsite specificity of the protease. In addition, we described the development of selective activity-based probes targeting KLK13 which, to our knowledge, is the first such attempt to analyze the presence of the active enzyme in biological samples.

Less comprehensive approaches to profiling KLK13 specificity were previously undertaken [18,20] and our results are in general agreement with these, while significantly extending the prior data. The most important determinant of KLK13 substrate specificity was located at the P_1_ position, exactly as expected for an S1 family protease. The preference for arginine at this subsite was clear, while lysine was recognized at the P_1_ position with significantly lower efficiency. Further analysis revealed a weaker selectivity at the P_4_-P_2_ positions. P_4_ favored Val and to a lesser extent Lys and Arg. This corresponds to the previous results of Borgoño et al., where Val was also the most preferred amino acid at that position. Nonetheless, our analysis of the P_4_ preference indicated Lys and Arg as the second and third most preferred residues, in contrast to Tyr as found in the work of Borgoño and colleagues. We also saw recognition of tyrosine at this subsite, but with low preference. This difference likely indicates the influence of the deconvolution scheme and substrate design on profiling results, especially where secondary, less pronounced specificities are concerned.

KLK13 specificity analysis at the P_3_ position revealed low selectivity. Arg was the most-preferred residue, but substrates containing multiple other residues were also efficiently hydrolyzed. Remarkably, Gly, Glu, Trp, Asp and Asn were incompatible with the P_3_ subsite—an observation unexpected at a subsite with otherwise poor selectivity. Such a result is again in partial agreement with previous reports. Borgoño et al. also identified Arg as the most preferred aminoacid at P_3_. Yet in their study, no other residues, with the exception of Lys, His, Val and Phe, were accepted at the P_3_ subsite. Andrade et al. also found Arg was one the most preferred residues at P_3_ subsite; however, in this instance, together with Lys Ile, Phe and to a lesser extent His, Val, Ala, Gln, Asn and Leu. Therefore, the preference for Arg at P_3_ is well established while secondary preference is less evident. Both we and Andrade et al. observed relatively broad specificity at this subsite, in contrast to Borgoño et al.’s report.

Due to the characteristic structure of the S2 subsite in KLK13 we expected limited specificity at the P_2_ position. Besides KLK1-3, KLK13 has the longest kallikrein loop among the KLK-family proteases, due to a characteristic 5-aa insertion. This is significant since the kallikrein loop shapes the S2 subsite. Indeed, our data support the prediction that a structurally restricted S2 subsite corresponds to extended specificity at this position. We found that Phe was favored at the P_2_ position, followed by Arg, Tyr and Leu. Other amino acids, including Lys, Met, Ser, His and Ala were recognized with much lower efficiency, and all other residues were disfavored at P_2_. Previous reports partially differ in their description of P_2_ preference, both between each other and with respect to our data. Borgoño et al. reported preferences for norleucine, Leu and for Phe, being the third most preferred residue, an observation accompanied by surprisingly low selectivity of KLK13 towards P_2_ residues in their study. In turn, Andrade et al. identified Arg and Ile as the most preferred residues at P_2_, followed by Lys, Leu and Phe. Although a certain consensus is evident, with Phe and Leu being reported among the most preferred residues in all three experimental setups, obvious differences are also noted. Our data are in better agreement with Andrade et al., indicating Arg among the preferred residues, a finding in contrast to Borgoño et al.’s report, where Arg was clearly disfavored. These differences are difficult to explain with substrate design only and may suggest certain cooperativity of the binding sites.

Analysis of specificity of KLK13 at primed positions was attempted only by Andrade et al. [20], although in a relatively less extensive setup. A series of peptides was synthesized, covering only 13 amino acid residues (out of 20 natural amino acids) in P_1_′ and P_2_′ positions, respectively. Our analysis was based on a more unbiased approach, where full libraries covering 20 residues were constructed for P_1_′, P_2_′ and P_3_′ positions. Moreover, the substrates used by Andrade et al. were based on an a priori selected constant non-primed region, while ours used a sequence of the most kinetically preferred substrate of KLK13. We found that KLK13 displayed a significant preference towards Ser in P_1_′ position, however Ala, His, Arg, Lys, Asn and Gln were also accepted with 30% efficacy, compared to the optimal sub-library containing Ser residue. This indicated limited selectivity of KLK13 in the P_1_′ position, in general agreement with previous reports, which identified Lys, His, Asn and Ser among the preferred residues. Notable differences were observed in recognition of Phe, which was disfavored in our study while being identified as one of the preferred amino acids in the abovementioned report. On the other hand, we have identified Arg as a residue recognized with efficiency comparable to that of Lys in P_1_′, which is in contrast to the previously described limited recognition of Arg at this position. Again, such differences could be related to the different design of tested substrates at the P_4_-P_1_ subsites and thus, different subsite cooperation. Andrade et al. determined P_1_′ specificity using the substrates of the general design ABZ-KLR↓XSKQ-EDDnp, as opposed to ABZ-VRFR↓P_1_′X_2_X_3_-Tyr(3-NO_2_)-NH_2_ used in this study. The presence of additional P_4_ Val, optimal P_3_ Arg and, most importantly, a large P_2_ Phe residue in our substrate libraries could affect the cooperativity of subsites and result in the observed varied selectivity of the enzyme with different substrate design. Indeed, KLK-family members are characterized by similar structure and organization of the substrate binding cleft [23,24], yet differ significantly in specificity, a phenomenon partially explained by the apparent cooperativity of the substrate binding subsites (where small differences at particular subsites may result in significant differences in the overall preference). For example, prior analysis of KLK14 specificity indicated strong interaction between S3 and S4 binding pockets, allowing identification of a preferred substrate, which was hydrolyzed significantly better than substrates previously identified using PC-SCL and phage display [25].

Analysis of the P_2_′ position revealed moderate selectivity, with Thr, Ser, Ala and Val being the most frequently selected amino acids, with most other residues still recognized, and only Asp, Glu and Gln being clearly rejected. These findings confirm the results of prior studies indicating moderate selectivity of KLK13 in the P_2_′ position except for Asp-containing substrates which were completely excluded. The notable differences concerned only Arg, Leu, Phe and Gln being the most preferred residues reported in the Andrade et al. study. We again attribute this difference to substrate and deconvolution scheme design which has a significant effect especially at subsites with lower selectivity.

To our knowledge this report was the first to analyze P_3_′ specificity of KLK13. KLK13 did not show a stringent specificity at this position, with the majority of residues accepted with similar efficiency. The exceptions were certain hydrophobic residues which were clearly disfavored: Val, Leu, Ile, Phe.

Based on comparative structural analysis, interactions between KLK13 and the substrate at positions further than P_4_ and P_3_′ are not expected and therefore our study provides a full overview of KLK13 substrate specificity. Our specificity profiling is in general agreement with prior studies, though expands the previous findings in a consistent design over all the subsites. Certain differences in profiling results were observed, which are especially evident when secondary specificities are considered. The variation in results may be explained by significant differences in the experimental design. First, our KLK13 was produced in a eukaryotic expression system which provided mammal-like glycosylation within the core glycan. Borgoño et al. obtained the enzyme in *Pichia pastoris*, an approach which results in excessive, unnatural glycosylation, reaching a 1:1 ratio of glycan and protein. Andrade and colleagues used baculovirus-expressed KLK13, obtaining glycosylation similar to our enzyme (note that our profiling data corresponds better to that of Andrade et al., indeed indirectly suggesting the influence of glycosylation). In addition, the experimental conditions differed significantly between the assays, as high-concentration (1–1.5 M) sodium citrate was used as an activator by some authors (Andrade et al.). All the parameters—excessive glycosylation, presence of cosmotropic activators and different substrate library design—may have affected the apparent activity and/or specificity of the enzyme, particularly in the less specificity defined positions and especially the secondary specificities. Therefore, not surprisingly, primary specificity in critical positions (P_1_, P_2_, P_3_) determined in our study fully corroborates the findings of prior reports. In contrast, specificity at less defined P′ positions, and secondary specificities show more variability. Nonetheless, we believe that because of its unbiased nature our approach presents the optimal method for determination of the KLK13 specificity. This is supported by obtaining an optimal substrate, characterized by the k_cat_/K_m_ ≈ 10^7^ M^−1^·s^−1^, the best KLK13 substrate described to date.

Based on the determined optimal substrate structure, we designed a KLK13 inhibitor by incorporating chloromethylketone (CMK) as a warhead. The CMK-inhibitor was further used to design two variants of biotin-tagged activity-based probes, one with the label attached directly at the N-terminus and the second with the PEG linker between the peptide fragment and the label. All three inhibitors demonstrated similar efficiency, with IC_50_ in the low-micromolar range and k_ass_ in the range of ~0.5–2.5 × 10^2^ M^−1^·s^−1^, however the presence of biotin in close proximity to the peptide part (without the PEG linker) slightly decreased the efficiency of inhibition. We determined the optimal concentration of the probe (low micromolar range) suitable for specific labeling of the active form of KLK13 only (as opposed to pro-KLK13). We also estimated the detection limit of the ABP at around 15 ng of the purified KLK13. These values are indeed satisfactory since similar concentrations of papain-like proteinase-specific activity-based probes—DCG-04 and its derivatives—were used in numerous reports [26,27,28,29]. Similarly, the detection limit established here, although significantly higher compared to the optimized antibody-based techniques [30], is comparable to that reported for activity-based probes targeting other enzyme families [31]. Therefore, the KLK13-specific activity-based probe designed here, Bt-PEG-VPR-CMK, is suitable for application in research aimed at the elucidation of KLK13 biological functions. Nonetheless, further development of the probe is still desired. In applications involving biotin, the sensitivity of the assay is limited by the background signal due to ubiquitous presence of endogenously biotinylated proteins in biological samples. Further optimization of the probe, e.g., by incorporation of a radioactive isotope label, would likely increase the sensitivity. In parallel, development of ABPs specific to different kallikreins and labeled with fluorophores of different spectral properties, would allow simultaneous detection of KLK-derived activities in complex samples.

Our probes efficiently detected KLK13 activity in complex biological samples, with low background in the analyzed cell lysates, culture medium and saliva. Notably, a weak staining of a lower molecular weight product in PMN-derived lysates was observed. This may indicate non-specific binding of the probe, yet it is worth noting that KLK13 expression was indeed detected in human PMN cells [32] and observed mass differences may reflect the presence of different isoforms of KLK13, which remains to be determined experimentally.

KLK13 was detected in all samples after spiking with the recombinant enzyme, confirming the probe stability and reactivity in complex biological samples. Additionally, the presence of KLK13 was detected in non-concentrated culture medium of the KLK13-expressing LEXSY strain, indicating good kinetics of the probe. These promising results open avenues for further research aimed at the elucidation of KLK13 activity in tissues. Similar approaches with cathepsin-specific ABPs allowed for the detailed description of CatB, L and S trafficking and activation in cell-based models of thyroid epithelium [33]. Most importantly, the availability of cathepsin-specific activity-based probes allowed for the analysis of their activity in the tumor environment in vivo [34], an exciting perspective, given the long-postulated role of kallikreins in the tumor progression, development and metastasis [35,36]. Herein, we have presented the first tool allowing for similar analysis of the kallikrein-related peptidase 13.

In conclusion, this report provides a first unbiased analysis of KLK13 protease specificity using optimized substrate libraries spanning both non-primed and primed subsites. We developed a highly-sensitive substrate (ABZ-Val-Arg-Phe-Arg-Ser-Thr-Gln-Tyr(3-NO_2_)-NH_2_) suitable for determination of KLK13 activity, characterized by k_cat_/K_m_ ≈ 10^7^ M^−1^·s^−1^. Even more importantly we delivered a validated, KLK13-optimized activity-based probe with the biotin tag. Both materials provided are valuable tools in protease research. The probes are active and suitable for detection of KLK13 in complex biological samples, hence opening new possibilities for further research on the biological role of KLK13 protease.

## 4. Materials and Methods

### 4.1. Expression and Purification of Recombinant Human KLK13

Total RNA was isolated from human airway epithelial (HAE) cells, cDNA was synthesized with the High-Capacity cDNA Reverse Transcription Kit (Thermo Fisher Scientific, Warsaw, Poland) and used as a template for proKLK13 gene amplification. To modify the product in a way suitable for cloning, a 3-step PCR was performed with primers (Forward1: 5′-GACGACGACAAGCTTGGTGACGTTGCCAATGCTGTG-3′, Forward2: 5′-CCATCATCACCACGACGACGACGACAAGCTTGGTGACGTTG-3′, Forward3: 5′-ATATCTAGACATCACCATCATCACCACGACGACGAC-3′ and Reverse 5′-ATAGCGGCCGCTTATTGTGGGCCCTTCAACCAT-3′) where the product of the first amplification was used as the template for the following PCR reaction and the same reverse primer was used for all of the reactions. The final product was cloned into the pLEXSY_I-blecherry3 plasmid (Jena Bioscience, Jena, Germany) with restriction sites *Not*I and *Xba*I. Correct insertion of the gene of interest into an open reading frame and the presence of a signal peptide was confirmed by sequencing. Plasmid pLEX-proKLK13 was amplified in *Escherichia coli* DH5α (Thermo Fisher Scientific) with ampicillin selection followed by Midiprep (A&A Biotechnology, Gdynia, Poland) clean up and linearized with *Sal*I (Thermo Fisher Scientific). All preparations for transfection, selection and expression in the host *L. tarentolae* strain T7-TR were performed according to the Jena Bioscience protocol for inducible expression of recombinant proteins secreted to the medium (Jena Bioscience). In brief, the host cells were electroporated with linear DNA construct and stable clones were selected with an appropriate antibiotic. Expression of proKLK13 was induced with 15 µg/mL of tetracycline (BioShop, Burlington, Canada) over 3 days, protein was precipitated out of the media with 80% ammonium sulfate, and the precipitates were collected by centrifugation at 15,000 RCF for 30 min at 4 °C, suspended in 10 mM sodium phosphate pH 7.5 and dialyzed overnight at 4 °C against the same buffer. The protein was recovered on immobilized nickel ions (GE Healthcare, Warsaw, Poland) in the presence of 10 mM benzamidine and purified according to the resin manufacturer’s protocol. Obtained fractions were analyzed by SDS-PAGE in reducing conditions, fractions containing proKLK13 were concentrated with Vivaspin 2 (Sartorius, Göttingen, Germany) and the protein was further purified on Superdex S75 pg (GE Healthcare) in 20 mM Tris-HCl, 0.5 M NaCl, pH 7.5. Fractions containing proKLK13 were concentrated with buffer exchange to 50 mM, Tris-HCl, 150 mM NaCl, pH 7.5. After purification and self-activation at 37 °C for 24 h, KLK13 was active-site titrated with recombinant LEKTI/SPINK6 as described by Kantyka et al. (2011) [37].

### 4.2. Individual Peptide and Peptide Library Synthesis

Individual substrates were synthesized manually on TentaGel S RAM resin (substitution level of 0.25 meq/g; RAPP Polymere, Tübingen, Germany) as the solid support. The Fmoc/tBu procedure was used. Coupling was carried out using an equimolar mixture of protected amino acid residue, DIPCI (*N*,*N*′-diisopropylcarbodiimide) and HOBt (1-hydroxybenzotriazole).

Peptide libraries were synthesized by the mixing-portioning method [22]. Specifically, 17.7 g of the solid support (TentaGel S RAM resin, RAPP Polymere, Tübingen, Germany) was used to synthesize the non-prime, ANB-based library. After the removal of Fmoc protecting groups with 20% piperidine in DMF/NMP (1:1, *v*/*v*), the resin-bound amine group was acylated with 5-amino-2-nitrobenzoic acid (ANB) using the *N*,*N*,*N*′,*N*′-tetramethyl-*O*-(benzotriazol-1-yl)uranium tetrafluoroborate (TBTU)/4-dimethylaminopyridine (DMAP). Briefly, two equivalents of ANB and two equivalents of TBTU/DMAP were dissolved in DMF and added to the resin. After 30 s, four equivalents of *N*,*N*-diisopropylethylamine (DIPEA) were added. The whole mixture was stirred for 3 h. The solution was filtered, and the resin was washed extensively with DMF. To achieve a complete resin substitution the procedure was repeated three times. Then, the first Fmoc-protected amino acid was conjugated to ANB using a POCl_3_/pyridine system [38]. After deprotection all subsequent Fmoc-protected amino acids were conjugated using the DIPCI/HOBt coupling system. A threefold excess of the reagents was applied, relative to the active resin sites. Finally, the Boc protected 2-aminobenzoic acid was introduced as the N-terminal group. The library of internally quenched ABZ/Tyr(3-NO_2_) peptides was synthesized as described above using 15.1 g TentaGel S RAM resin and starting with Fmoc-protected 3-nitro-l-tyrosine.

Following synthesis, the peptides were cleaved from the resin using a trifluoroacetic acid (TFA)/phenol/triisopropylsilane/H_2_O mixture (88:5:2:5, *v*/*v*) [39]. The purity of the peptides was evaluated on a RP-HPLC Jasco LC System (Jasco, Tokyo, Japan) equipped with Supelco Wide Pore C8 column (8 × 250 mm) and ultraviolet-visible (UV-VIS, 226 nm) and fluorescent detectors (excitation 320 nm, emission 450 nm). A linear gradient from 10% to 90% B within 40 min was applied (A: 0.1% TFA in water; B: 80% acetonitrile in A). Mass spectra were recorded using a Biflex III MALDI TOF mass spectrometer (Bruker, Karlsruhe, Germany) on α-cyano-4-hydroxycynnamic acid (CCA) or 2,5-dihydroxybenzoic acid (DHB).

### 4.3. Activity-Based Probe Synthesis

The activity-based probes were synthesized in a two-step procedure. First, the peptide fragments (Ac-Val-Arg-Phe-OH, Bt-Val-Arg-Phe-OH and Bt-PEG-Val-Arg-Phe-OH) were obtained manually on a 2-chlorotrityl resin as the solid support. Peptides were cleaved from the resin with side-chain protecting groups [40]. One molar equivalent of each peptide was dissolved in DMF and 1.1 molar equivalent of l-arginine chloromethylketone, 2 molar equivalents of DEPBT (3-(diethoxyphosphoryloxy)-1,2,3-benzotriazin-4(3H)-one) and 3 molar equivalents of DIPEA were added. The reaction mixture was stirred for 6 h. The organic solvent was evaporated. The side-chain protecting groups were removed using trifluoroacetic acid (TFA)/phenol/triisopropylsilane/H_2_O mixture (88:5:2:5, *v*/*v*) [39].

### 4.4. Library Deconvolution

The specificity of KLK13 at nonprime subsites was evaluated using an ABZ/ANB library and the enzyme in concentrations ranging from 8.32 × 10^−8^ to 1.75 × 10^−8^ M. Deconvolution of the primed subsite library was carried out using KLK13 at concentrations from 1.25 × 10^−8^ to 1.25 × 10^−9^ M. Deconvolution was performed using the iterative method in solution [41]. Freeze-dried samples of each sub-library were dissolved in dimethyl sulfoxide (DMSO) to the final concentration of 5 mg/mL. 20 mL of each tested sub-library was supplemented with an assay buffer (180 μL; 50 mM Tris-Cl (pH 7.5) containing 1 mM EDTA and 0.5 μM heparin [20] and with the enzyme. All measurements were performed using the Omega plate reader (BMG Labtech, Ortenberg, Germany). Hydrolysis was monitored for 30 min at 37 °C by the ANB absorbance at 410 nm (for the non-primed library) or at ABZ excitation wavelength of 320 nm and emission at 450 nm (for the primed library).

The site of hydrolysis was determined after each step of deconvolution of primed site library and for both final substrates (**1** and **2**) by RP-HPLC monitored by fluorescence detection and MS.

### 4.5. Determination of Kinetic Parameters

Kinetic parameters (Michaelis constants (K_M_) and catalytic constants (k_cat_)) were determined at a KLK13 concentration of 1.25 × 10^−9^ M. All measurements were performed in triplicate and the systematic error, expressed as a standard deviation, did not exceed 20%. The specificity constant (k_cat_/K_M_) was calculated from k_cat_ and K_M_. The details of kinetic studies and the method of calculating kinetic parameters have been described previously [42]. 

### 4.6. Determination of Detection Limit

The detection limit was established by analyzing the correlation between an increase in absorbance/fluorescence and enzyme concentration at constant quantities of each, ABZ-Val-Arg-Phe-Arg-ANB-NH_2_ (Substrate **1**) and ABZ-Val-Arg-Phe-Arg-Ser-Thr-Gln-Tyr(3-NO_2_)-NH_2_ (Substrate **2**).

### 4.7. pH Dependence of KLK13 Activity

The pH dependence of KLK13 (1.25 nM) was established at 37 °C using substrate **2** (6.15 µM) in following buffers: citric acid/sodium citrate (pH 3), sodium acetate/acetic acid (pH 4 and 5), MES (pH 6), MOPS (pH 7), HEPES (pH 8) and TRIS (pH 9 and 10).

### 4.8. Selectivity Assessment

Substrate **2** (6.15 μM) was incubated with tested kallikreins (KLK1, KLK2, KLK3, KLK4, KLK6, KLK8, KLK12, KLK13) and cathepsins (CatX, CatL, CatS, CatD, CatH, CatB), all at a concentration of 1.25 nM, at 37 °C in 50 mM Tris-Cl (pH 7.5) buffer containing 1 mM EDTA and 0.5 μM heparin. Proteolytic activity was assayed as an increase in fluorescence at 450 nm.

### 4.9. Determination of Inhibitory Activity

The kinetic parameters of inhibition were determined using a progress curve model for irreversible inhibition, as described previously [43,44]. Briefly, a series of concentrations (0–50 µM) of activity-based probes (ABPs) obtained in this study (Ac-Val-Arg-Phe-Arg-CMK, Bt-Val-Arg-Phe-Arg-CMK and Bt-PEG-Val-Arg-Phe-Arg-CMK) was mixed with a substrate ABZ-Val-Arg-Phe-Arg-Ser-Thr-Gln-Tyr(3-NO_2_)-NH_2_, (Substrate **2**, final concentration 10 µM). Upon the addition of KLK13 (final concentration 10 nM), the fluorescence of the hydrolyzed substrate was monitored in each sample at excitation and emission wavelengths of 320 and 450 nm, respectively, for 1 h using a microplate reader Gemini EM (Molecular Devices, Berkshire, UK). The reactions were carried out in 200 µL of final volume of 50 mM Tris-Cl (pH 7.5) containing 1 mM EDTA and 0.5 μM heparin at 37 °C. Progress curves were fitted to the resulting data points and then k_obs_ derived from each curve was plotted against inhibitor concentration. Final values of k_ass_ were calculated by linear regression and presented as mean ± SD of three independent replicates. IC_50_ values were determined by incubating increasing amounts of each inhibitor with 10 nM KLK13 and linear regression to the initial fragment of the resulting curve.

### 4.10. Detection of KLK13 Using ABPs

Purified active KLK13 (250 ng) was preincubated with increasing concentrations of Bt-PEG-VRFR-CMK or Bt-VRFR-CMK in TNET buffer (0.1 M Tris-HCl, 150 mM NaCl, 5 mM EDTA, 0.05% Tween-20, pH 7.5) for 1 h at 37 °C. Samples were analyzed by SDS-PAGE followed by wet electrotransfer onto a PVDF membrane (GE Healthcare). Membranes were blocked in 5% BSA in TBS buffer (50 mM Tris-HCl, 0.5 M NaCl, pH 7.5) with 0.1% Tween overnight, at 4 °C, followed by incubation with 1:10,000 diluted Streptavidin-HRP (Sigma Aldrich, S2438, Poznan, Poland) for 2 h at RT. After intensive washing with TBS buffer with 0.1% Tween, the signal was developed with Pierce ECL WB substrate (Thermo Fisher Scientific) and visualized manually using AGFA films (Agfa HealthCare, Mortsel, Belgium). To establish the detection limit, decreasing amounts of active KLK13 (ranging from 3.9-500 ng) were preincubated with 1 µM Bt-PEG-VRFR-CMK in TNET buffer for 1 h at 37 °C and visualized by streptavidin-HRP Western blot as described above.

### 4.11. Detection of KLK13 in Biological Samples

The activity of KLK13 was analyzed in 3-day conditioned LEXSY media using strains stably transfected with empty pLEX and pLEXpKLK13 plasmids. Undiluted culture supernatant (27 µL) was preincubated with or without 1 µM Bt-PEG-VRFR-CMK probe for 1 h at 37 °C, followed by Western blot analysis.

Saliva, freshly collected from healthy donors, was clarified by centrifugation and diluted 2-fold with sterile TNET buffer. The samples were spiked with 250 ng of KLK13 and incubated with 1 µM tested activity-based probe for 1 h at 37 °C, followed by Western blot analysis (final concentration of saliva in samples was 25%).

Cell lysates were prepared as follows: neutrophils were isolated from the blood of healthy donors [45] and lysed in RIPA buffer (25 mM Tris pH 7.5, 150 mM NaCl, 1% Nonidet P-40, 0.1% SDS, 0.5% sodium deoxycholate)

Human telomerase immortalized keratinocytes (TIGK, ATCC^®^ CRL-3397^TM^, LGC Standards, Teddington, UK) were cultured in KBM-Gold^TM^ (Lonza, Warsaw, Poland) keratinocyte basal medium supplemented with Single Quotes^TM^ (Lonza, Warsaw, Poland) at 37 °C and 5% CO_2_. Cells were collected by trypsin digest and washed with PBS. Finally, cells were resuspended in TNET buffer, lysed on ice with a hand sonicator (amplitude 60%, 20 cycles, 1 s pulse, 5 s break; UP50H Ultrasonic Processor, Hielscher, Teltow, Germany) and cleared by centrifugation. Cell lysates (30 µg of PMN and 5 µg of TIGK lysate protein content, respectively) were spiked with 250 ng of recombinant KLK13 and incubated with 1 µM specific ABP for 1 h 37 °C. All samples were analyzed with Western blot and compared to the unspiked and unlabeled controls.

## Figures and Tables

**Table 1:** Summary of P_4_-P_3_′ residues most preferred by kallikrein 13 (KLK13) as determined by this and prior studies. All percentage values were calculated and compared from each publication separately. Activity on the most preferred residue was considered as 100% and the following amino acids were then adjusted accordingly to the highest value for each position. Only amino acids with a threshold over 50% are listed, with the exception of the P_3_′ values where an 80% threshold was used due to low selectivity at that position (*). n.d.—not determined.

	This Work	Borgoño et al. [18]	Andrade et al. [20]
**P_4_**	V (100%), K (62%)	V (100%), Y (58%)	n.d.
**P_3_**	R (100%), A (58%)	R (100%)	K (100%), I (96%) R (80%), F (74%), V (56%), H (56%)
**P_2_**	F (100%)	M (100%), L (80%), F (65%)	R (100%), I (94%), K (59%)
**P_1_**	R (100%)	R (100%)	R (100%)
**P_1_′**	S (100%)	n.d.	H (100%), K (61%)
**P_2_′**	T (100%), A (79%), S (79%), V (54%)	n.d.	R (100%)
**P_3_′**	Q (100%), G (93), S (89%), W (84%), N (82%) *	n.d.	n.d.

**Table 2:** Kinetic parameters of the selected KLK13-specific substrates. MW_exp_ represent the MS-TOF measured average mass of the compounds.

Substrate	MW_exp_	K_M_ (µM)	k_cat_ (s^−1^)	k_cat_/K_M_ ([M^−1^·s^−1^])
ABZ-VRFRST**Q**-Tyr(3-NO_2_)-NH_2_ (**2**)	1247.0	10.0 ± 1.9	96.43 ± 7.15	(9.64 ± 1.08) ×10^6^
ABZ-VRFRST**E**-Tyr(3-NO_2_)-NH_2_	1249.1	24.7 ± 5.8	91.11 ± 11.61	(3.69 ± 0.40) ×10^6^
ABZ-VRFRST**G**-Tyr(3-NO_2_)-NH_2_	1175.9	19.7 ± 3.0	169.40 ± 12.40	(8.62 ± 0.37) ×10^6^
ABZ-VRFRST**S**-Tyr(3-NO_2_)-NH_2_	1220.7	14.7 ± 2.2	113.12 ± 6.87	(7.71 ± 0.67) ×10^6^
ABZ-VRFR-ANB-NH_2_ (**1**)	858.8	17.8 ± 2.8	94.36 ± 3.05	(5.31 ± 0.34) ×10^6^

## Supplementary Materials

Supplementary materials can be found at https://www.mdpi.com/1422-0067/20/7/1557/s1.

## Abbreviations

KLK	Kallikrein-like proteinase
ABP	Activity-based probe
PEG	Polyethylene glycol
LEXSY	*Leishmania* Expression System (LEXSY)
HAE	Human airway epithelial
ANB	5-Amino-2-nitrobenzoic acid
TBTU	*N,N,N*′,*N*′-Tetramethyl-*O*-(benzotriazol-1-yl)uranium tetrafluoroborate
DMAP	4-Dimethylaminopyridine
DIPEA	*N*,*N*-Diisopropylethylamine
DIPCI	*N*,*N*′-Diisopropylcarbodiimide
HOBt	1-Hydroxybenzotriazole
ABZ	Albendazole
TFA	Trifluoroacetic acid
CCA	α-Cyano-4-hydroxycynnamic acid
DHB	2,5-Dihydroxybenzoic acid
DEPBT	3-(Diethoxyphosphoryloxy)-1,2,3-benzotriazin-4(3H)-one
TIGK	Telomerase immortalized keratinocytes
