# Supplementary material for: Development of Chemical Tools to Monitor Human Kallikrein 13 (KLK13) Activity
Source: Int J Mol Sci. 2019 Mar 28;20(7):1557. doi: 10.3390/ijms20071557 (PMC6479877; doi:10.3390/ijms20071557)
Supplement: Supplementary file 1 [file ijms-20-01557-s001.pdf]

International Journal of
Molecular Sciences

MDPI

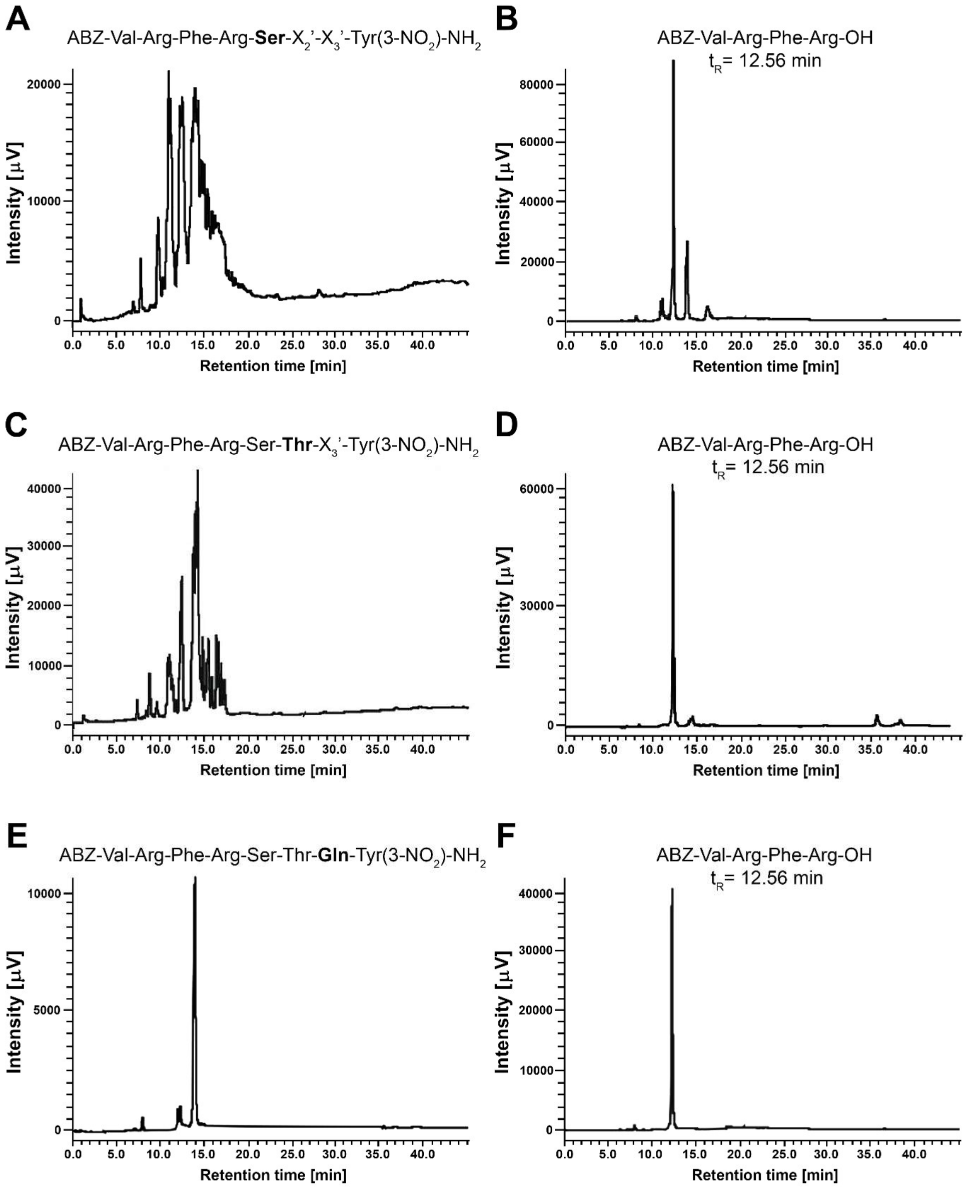

G

Library	Measured MW	Theoretical MW
ABZ-Val-Arg-Phe-Arg-Ser-X_2'-X_3'-Tyr(3-NO_2)-NH_2	1104.18 - 1362.5	1105.17 - 1363.49
ABZ-Val-Arg-Phe-Arg-Ser-Thr-X_3'-Tyr(3-NO_2)-NH_2	1149.26 - 1278.42	1149.22 - 1278.38
ABZ-Val-Arg-Phe-Arg-Ser-Thr-Gln-Tyr(3-NO_2)-NH_2	1220.34	1220.3

Figure S1. Sites of KLK13 hydrolysis in libraries used for primed site profiling. (**A**) Library with general formula ABZ-Val-Arg-Phe-Arg-Ser-X_2′-X_3′-Tyr(3-NO_2); (**B**) library shown in panel A after incubation with KLK13; (**C**) library with general formula ABZ-Val-Arg-Phe-Arg-Ser-Thr-X_3′-Tyr(3-NO_2); (**D**) library shown in panel C after incubation with KLK13; (**E**) peptide ABZ-Val-Arg-Phe-Arg-Ser-Thr-Gln-Tyr(3-NO_2); (**F**) peptide shown in panel E after incubation with KLK13. ABZ fluorescence was monitored at the excitation and emission wavelengths of 320 and 450 nm, respectively. In panels C, D and F—retention time and MS identification of the major product are given above the chromatogram. Note that all tested substrates generated the same ABZ-Val-Arg-Phe-Arg-OH (t_R12.56 min) product signifying KLK13 catalyze hydrolysis at Arg-Ser peptide bond, regardless the identity of the variable regions. (**G**) MS spectra of the libraries and optimized substrate **2** were recorded and confirmed the identity of the investigated compounds.

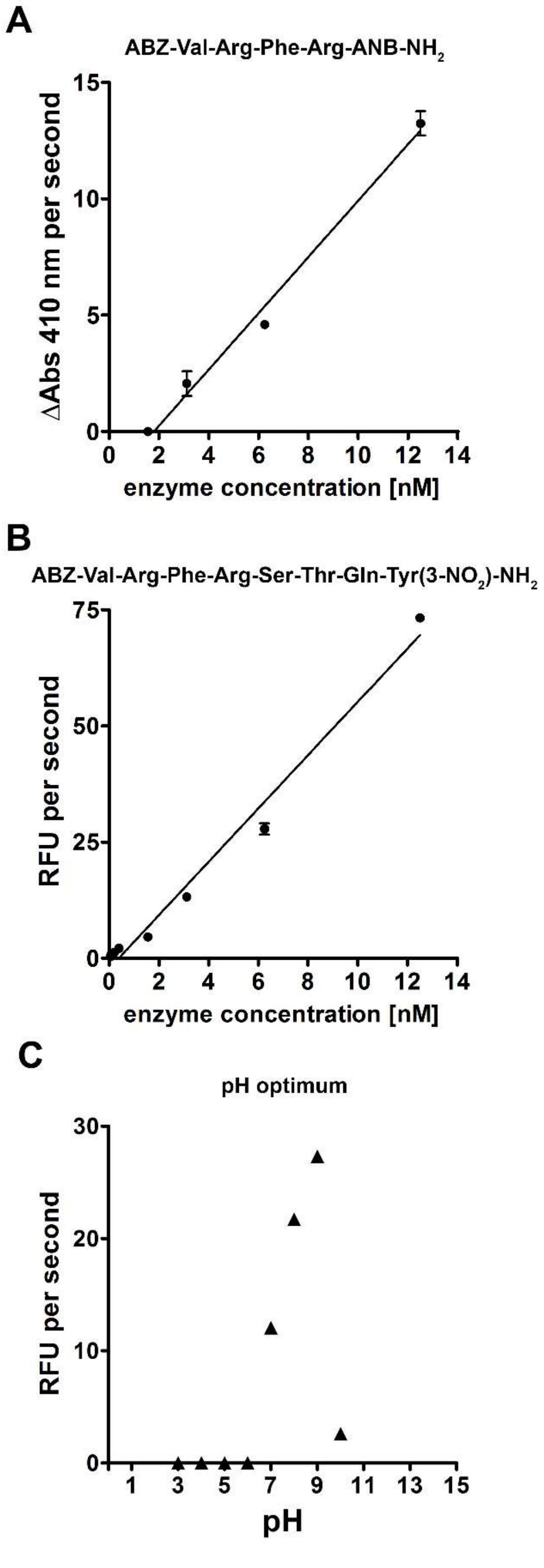

Figure S2. Detection limit and pH optimum of KLK13 as determined for the optimized substrates. Substrates **1** (**A**) and 2 (**B**) were titrated with decreasing amounts of KLK13 in 50 mM Tris-Cl (pH 7.5) buffer containing 1 mM EDTA and 0.5 μM heparin at 37 °C. (**C**) pH dependence of KLK13. Processing of ABZ-Val-Arg-Phe-Arg-Ser-Thr-Gln-Tyr(3-NO_2)-NH_2 by KLK13 was monitored at pH range of 3 to 10 (citric acid/sodium citrate, sodium acetate/acetic acid, MES, MOPS, HEPES and TRIS buffers). Reaction progress was monitored by fluorescence detection at the excitation and emission wavelength 310 and 450 nm, respectively.
